# Determinants of Homologous Recombination Deficiency in Pancreatic Cancer

**DOI:** 10.3390/cancers13184716

**Published:** 2021-09-21

**Authors:** Max M. Wattenberg, Kim A. Reiss

**Affiliations:** 1 Division of Hematology-Oncology, Department of Medicine, Perelman School of Medicine, University of Pennsylvania, Philadelphia, PA 19104, USA; max.wattenberg@pennmedicine.upenn.edu; 2 Abramson Cancer Center, Perelman School of Medicine, University of Pennsylvania, Philadelphia, PA 19104, USA

**Keywords:** homologous recombination deficiency, pancreatic cancer, DNA damage repair

## Abstract

**Simple Summary:**

A subset of patients with pancreatic cancer demonstrate heightened response rates and prolonged survival to platinum chemotherapy and PARP inhibitors. Deficient homologous recombination (HR), a critical DNA repair program, is a major driver of this susceptibility. Furthermore, the clinical impact of mutations in distinct HR genes is variable and is modified by diverse tumor intrinsic and extrinsic factors. In this review, we discuss the determinants of homologous recombination deficiency (HRD) in pancreatic cancer. We also highlight emerging methods for identifying and inducing HRD in cancer.

**Abstract:**

Pancreatic cancer is a treatment-resistant malignancy associated with high mortality. However, defective homologous recombination (HR), a DNA repair mechanism required for high-fidelity repair of double-strand DNA breaks, is a therapeutic vulnerability. Consistent with this, a subset of patients with pancreatic cancer show unique tumor responsiveness to HR-dependent DNA damage triggered by certain treatments (platinum chemotherapy and PARP inhibitors). While pathogenic mutations in HR genes are a major driver of this sensitivity, another layer of diverse tumor intrinsic and extrinsic factors regulate the HR deficiency (HRD) phenotype. Defining the mechanisms that drive HRD may guide the development of novel strategies and therapeutics to induce treatment sensitivity in non-HRD tumors. Here, we discuss the complexity underlying HRD in pancreatic cancer and highlight implications for identifying and treating this distinct subset of patients.

## 1. Introduction

Pancreatic cancer is a therapy-resistant malignancy and a leading cause of cancer-related mortality [1]. Historically, treatment for pancreatic cancer has largely been delivered uniformly, without regard to underlying tumor biology [2,3]. However, with the approval of the poly (ADP-ribose) polymerase inhibitor (PARPi) olaparib as maintenance for patients with pancreatic cancer and a germline *BRCA* variant, there is renewed fervor to broaden and better define homologous recombination deficiency (HRD), develop clinically usable assays to capture this population, design second-generation therapies and induce HRD in wildtype tumors.

The genes *BRCA1* and *BRCA2* code for critical protein members of homologous recombination (HR), a conservative DNA repair mechanism involved in the repair of DNA double-strand breaks (DSBs) [4,5]. HRD tumor cells are defined by genomic instability, which facilitates mutagenesis and tumorigenesis [4,5]. As such, pathogenic germline mutations in *BRCA* genes are associated with heritable cancer syndromes, predisposing to multiple solid tumors, including breast, ovarian, prostate and pancreatic cancer [6,7]. HRD tumor cells are characterized by a striking sensitivity to platinum chemotherapy and PARPi [4,5], but HRD and responsiveness to platinum chemotherapy/PARPi are not solely restricted to *BRCA* mutant tumors [8]. Mutations in some non-*BRCA* HR genes are also associated with HRD, although not all non-*BRCA* HR gene mutations confer the same clinical impact [9]. Further, it is increasingly recognized that even in tumors with bona fide HR gene mutations, additional tumor intrinsic and extrinsic factors can modulate the phenotype of sensitivity to platinum chemotherapy/PARPi (HRD phenotype). Thus, several criteria must be met for a tumor to display HRD that results in sensitivity to platinum chemotherapy/PARPi, including (i) functionally meaningful disruption of HR, (ii) lack of tumor intrinsic HR bypass mechanisms and (iii) a distinct tumor microenvironment.

To provide an overview of the current mechanistic understanding of HRD, we will discuss relevant pre-clinical and clinical studies that define tumor intrinsic (genetic) and extrinsic (tumor microenvironment) factors that contribute to platinum chemotherapy and PARPi sensitivity. A major challenge moving forward is how to more precisely identify clinically relevant biomarkers that are truly predictive for platinum and PARPi sensitivity. We will review current and emerging testing strategies to identify HRD. Another unsolved problem is that only a minority of pancreatic cancer harbors mutations in clinically relevant HR genes [10]. We will discuss novel therapeutic strategies aimed at inducing HRD in non-HRD and platinum/PARPi-resistant tumors. Taken together, our review highlights a distinct and clinically relevant subset of patients with pancreatic cancer and provides a framework for the development of testing and treatment strategies aimed at this population.

## 2. Tumor Intrinsic and Extrinsic Determinants of HRD

**Figure 1:**
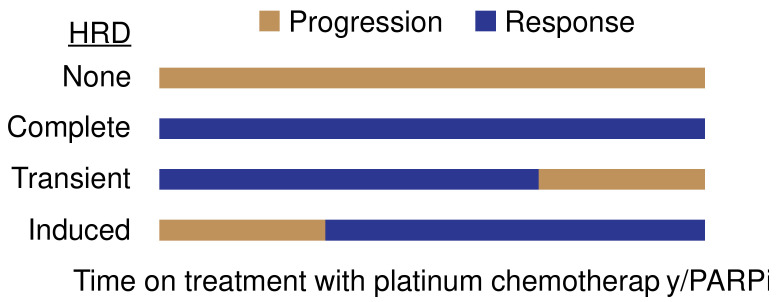
Clinical scenarios of homologous recombination deficiency (HRD). PARPi, poly (ADP-ribose) polymerase inhibitor.

Patients show distinct patterns of response to platinum chemotherapy/PARPi treatment (Figure 1). For example, patients can show (i) no response to therapy (lack of HRD), (ii) complete and durable response to therapy (complete HRD), (iii) initial response to therapy followed by progression (transient HRD) or (iv) initial progression followed by response to therapy, which may occur in the setting of inducing HRD with a novel therapy. Defining the factors that shape sensitivity to platinum chemotherapy/PARPi is crucial to inform strategies to induce and maintain HRD. Here, we discuss the key biological determinants of HRD, which include (i) stereotyped mutations in specific genes associated with DNA repair, (ii) activity of non-HR cell intrinsic pathways and (iii) an immune permissive tumor microenvironment (Table 1).

### 2.1. Homologous Recombination and Sensitivity to Platinum Chemotherapy and Poly (ADP-Ribose) Polymerase Inhibitors

HR is a conservative DNA repair mechanism, which mediates repair of DNA double-stranded breaks (DSBs) [11]. Moreover, HR proficiency is a major cell intrinsic determinant of sensitivity to platinum chemotherapy and PARPi [12]. HR consists of defined steps, for which the molecular biology has been previously reviewed in detail [11,13,14]. In order to highlight the physiologic contributions of HR proteins of clinical interest, we will describe two critical steps of HR, end resection and strand invasion. The generation of 3′ single-strand DNA (ssDNA) overhangs, via end resection, is a key early step in HR. Upon development of a DNA DSB, the MRE11-RAD50-NBS1 (MRN) complex rapidly accumulates at sites of DNA damage [11,15]. Among several functions, MRN provides endonuclease activity, which mediates cleavage of 5′ ends internal to break ends, generating a 3′ tail. Following MRN activity, further resection is mediated by distinct HR members, including the exonuclease EXO1 and a complex containing the endonuclease DNA2 [15]. Furthermore, MRN recruits ATM, which phosphorylates numerous protein targets involved in cell cycle control and DNA repair, including BRCA1 [16,17]. Notably, additional kinases, including CHK2 and ATR, can also phosphorylate BRCA1 in the correct context [18]. BRCA1, which complexes with BARD1, plays multiple roles in HR, promoting resection by interacting with MRN, in addition to interacting with downstream HR members [19,20]. In this regard, BRCA1 directly interacts with PALB2, which bridges to BRCA2, thus participating in recruitment of BRCA2 and, in part, promoting initiation of strand invasion [18,19,21]. BRCA2 mediates the loading of RAD51 onto ssDNA, driving creation of a nucleoprotein filament, which searches for and invades template dsDNA [22]. RAD51 paralogues (RAD51B, RAD51C, RAD51D, XRCC2 and XRCC3) also assist in recruiting RAD51 for nucleoprotein formation [11]. Following invasion, DNA synthesis and ligation are performed, and the DNA structure is resolved [23]. Given the complexity of HR, mutations in multiple genes (e.g., *BRCA* vs. non-*BRCA*) can drive increased sensitivity to platinum chemotherapy and PARPi.

#### 2.1.1. Platinum Chemotherapy in Pancreatic Cancer

In unselected patients with pancreatic cancer, platinum chemotherapy shows variable activity. For example, oxaliplatin in combination with 5-FU, leucovorin and irinotecan (FOLFIRINOX) is approved by the US Food and Drug Administration (FDA) for first-line treatment of pancreatic cancer, while cisplatin in combination with gemcitabine failed to improve outcomes as compared to gemcitabine monotherapy in an unselected patient population [3,24]. In contrast, patients with HRD pancreatic cancer show striking clinical benefit and prolonged overall survival (OS) to platinum-based treatments [25,26,27,28]. HR-deficient tumor cells fail to resolve platinum-induced inter- and intra-strand DNA cross-links and accumulate DNA damage, eventually becoming non-viable [29]. It should be noted that oxaliplatin differs from cisplatin/carboplatin in regard to the level of DNA adduct formation, and thus is hypothesized to have reduced activity in the setting of HRD [30,31]. However, retrospective clinical data suggest similar activity of oxaliplatin and cisplatin in patients with germline *BRCA* and *PALB2* mutations [27,32], though this has not been prospectively validated. It is possible that the higher dose intensity of oxaliplatin as compared to cisplatin (85 vs. 25 mg/m^2^) that is used in clinical practice accounts for the discrepancy between pre-clinical and clinical data regarding oxaliplatin activity in HRD tumors.

#### 2.1.2. Mutations in *BRCA* Genes and Sensitivity to Platinum Chemotherapy

Mutations in *BRCA1, BRCA2* and *PALB2*, which produces a BRCA2-associated protein and is considered ‘*BRCA*-like’, are the prototypes of HRD [25]. A phase II clinical trial comparing gemcitabine plus cisplatin with or without the PARPi veliparib in patients with germline *BRCA* or *PALB2* mutations prospectively confirmed response rates to platinum chemotherapy approaching 70% in this population [26]. Notably, there was no significant clinical benefit to the addition of veliparib over platinum-chemotherapy alone and increased hematologic toxicity with the addition of veliparib was seen [26]. Importantly, of available classes of chemotherapy, platinum salts are uniquely effective for the treatment of *BRCA* mutant pancreatic cancer. In this regard, based on retrospective data, OS with platinum chemotherapy is longer than with non-platinum chemotherapy in patients with *BRCA* mutant pancreatic cancer [33]. Although retrospective analysis suggests similar OS among patients with *BRCA* mutant pancreatic cancer and patients with sporadic cancer when treated with non-platinum chemotherapy, *BRCA* mutations in pancreatic cancer may be prognostic as well as predictive of improved outcomes, regardless of therapy [34].

Retrospective data highlight the conditionality of HRD in the setting of mutant *BRCA*. For example, platinum chemotherapy administration in the first line of treatment as compared to later lines is associated with improved clinical outcomes [27]. Additionally, loss of heterozygosity (LOH) is critical for platinum and PARPi sensitivity. Recent data suggest that in patients with pancreatic cancer and a pathogenic germline variant in *BRCA*, up to 12% may not demonstrate HRD, when evaluated by genomic HR classifiers. These patients plausibly retain a wildtype allele and therefore would not be predicted to have pronounced sensitivity to platinum chemotherapy or PARP inhibitors [9,35]. In keeping with this, biallelic inactivation of HR genes is associated with longer OS, as compared to monoallelic mutations in patients treated with platinum chemotherapy [9]. Lastly, the impact of *BRCA* mutations on phenotype is dynamic in the face of therapy. In patients with *BRCA* mutant cancer, resistance to platinum chemotherapy is commonly associated with secondary reversion mutations, which restore the open reading frame of *BRCA,* thereby allowing transcription of an active BRCA protein [36]. Although reversion mutations as a resistance mechanism to platinum chemotherapy have mostly been described in patients with ovarian and breast cancer, a number of case reports and case series highlight the same scenario in patients with pancreatic cancer [9,37,38,39,40,41].

#### 2.1.3. Mutations in Non-*BRCA* HR Genes and Sensitivity to Platinum Chemotherapy

In contrast to the striking impact of *BRCA* gene mutations on tumor biology, the role of non-*BRCA* HR gene mutations in pancreatic cancer is more variable. One major challenge limiting robust clinical evaluation is the diversity and rarity of alternate HR gene mutations. As such, accruing to a clinical trial studying any one non-*BRCA* HR gene mutation would be restrictive, which has led to grouping of these patients in retrospective and prospective studies [25,28,42]. For example, a retrospective study found longer progression-free survival (PFS) for patients with pancreatic cancer and non-*BRCA* HR gene mutations (*ATM, BAP1, BARD1, BLM, BRIP1, CHEK2, FAM175A, FANCA, FANCC, NBN, RAD50, RAD51, RAD51C, RAD51D, RTEL1*) compared to patients without a known HR mutation when treated with platinum chemotherapy [43]. However, by combining patients with a variety of discrete mutations, clinically relevant positive or negative signals from one specific variant may be diluted, making it difficult to identify genophenotypic correlations. In line with this, although *ATM* and *CHEK2* mutations have been suggested to associate with increased response to DNA-damaging therapy, a recent analysis showed that *ATM* and *CHEK2* mutant pancreatic cancer does not show evidence of HRD by multiple genetic HR classifiers [9]. Taken together, although distinct non-*BRCA* HR mutations contribute to HRD, identifying patients for treatment with platinum chemotherapy/PARPi by specific mutation is not a sufficient patient selection method. Thus, a focus on alternative classifiers and multimodal testing strategies to identify HRD is critical. Later in the review, we will discuss in detail genetic and functional classifiers of HR and their potential for clinical use.

#### 2.1.4. Poly (ADP-Ribose) Polymerase Inhibitors in Cancer

The PARP protein family consists of multiple members and participates in diverse cellular processes via the post-translational modification PARylation [44]. In particular, PARP1, which is a major target of current PARPi, plays a role in DNA damage signaling, chromatin remodeling and replication fork stabilization [45]. Further, genetic loss or inhibition of PARP is synthetically lethal in *BRCA* mutant cells [46,47]. Currently, multiple small-molecule inhibitors of PARP, including olaparib, rucaparib, niraparib and talazoparib, have been approved by the FDA with indications for the treatment of breast (olaparib, talazoparib), ovarian (olaparib, rucaparib, niraparib), prostate (olaparib, rucaparib) and pancreatic cancer (olaparib) [48].

Two major mechanisms of action of PARPi-mediated cytotoxicity have been proposed. First, given that PARP1 has been implicated in DNA single-strand break (SSB) repair, inhibition of PARP has been suggested to allow accumulation of SSB followed by degeneration to DSB [49,50]. However, studies utilizing genetic ablation of PARP1 do not show increased frequencies of SSB, and ablation of XRCC1, which interacts with PARPi and is important for base excision repair, fails to show synthetic lethality with BRCA2 deficiency, challenging this mechanism [51,52]. More likely, PARPi acts to ‘trap’ PARP at the site of SSBs. The resulting PARP/DNA complex ultimately leads to replication fork collapse and DSB formation, requiring HR repair [53]. Consistent with this, others have suggested that the cytotoxic potential of distinct small-molecule inhibitors of PARP correlates with PARP trapping capacity [48]. Further, as discussed in detail in subsequent sections, in addition to tumor intrinsic mechanisms of cytotoxicity, PARPi trigger activation of the cyclic GMP–AMP (cGAMP) synthase (cGAS)— stimulator of interferon genes (STING) pathway inducing anti-tumor immunity [54]. Continued investigation into the tumor intrinsic and extrinsic mechanism(s) of action of PARPi will be critical to fully understand potential resistance pathways at play and identify rational combination strategies.

#### 2.1.5. Mutations in *BRCA* Genes and Sensitivity to Poly (ADP-Ribose) Polymerase Inhibitors

A signal for the use of PARPi for the treatment of *BRCA*-related pancreatic cancer was first identified in patients with relapsed disease [55,56,57]. Although clinical efficacy was limited in this patient population, patients with platinum-sensitive pancreatic cancer showed potential benefits, providing critical rationale for further testing of PARPi in the platinum-sensitive setting [56]. To this end, the randomized, phase III POLO (Pancreas Cancer Olaparib Ongoing) trial tested the role of olaparib as a maintenance treatment for patients with germline BRCA mutations and no progressive disease after at least 16 weeks of platinum-based therapy. Patients were randomized in a 3:2 fashion to receive either olaparib or a placebo pill. The study demonstrated a doubling of PFS (7.4 mo vs. 3.8 mo; HR 0.45) and led to the FDA approval of olaparib in this clinical setting [58]. The dual biomarker strategy (germline *BRCA* mutation and platinum sensitivity) used in the POLO trial reflects the complexity of identifying HRD. The maintenance PARPi paradigm has been confirmed and extended in a single-arm phase II clinical trial showing activity of rucaparib as a viable maintenance treatment in advanced pancreatic cancer. This second study included patients with somatic *BRCA2* and germline *PALB2* mutations [59]. The trial was notable for a PFS of 13.1 months, and an ORR of 41.7%, with responses seen in 3/6 patients with *PALB2* variants and in 1/2 patients with somatic *BRCA* variants. High disease burden was associated with poor outcomes, suggesting that additional tumor-specific biology interacts with HR mutations to drive HRD in pancreatic cancer [59].

#### 2.1.6. Mutations in Non-BRCA Genes and Sensitivity to Poly (ADP-Ribose) Polymerase Inhibitors

The efficacy of PARPi in patients with non-*BRCA* HR mutations has thus far been somewhat disappointing. A recently published study combining two parallel phase 2 clinical trials assessed olaparib monotherapy in 24 patients with previously treated pancreatic cancer and non-*BRCA* HR gene alterations (somatic *BRCA*, germline or somatic *ATM, PALB2, ARID1A, PTEN, RAD51, CCNE* and *FANCB*). Two responses (8%) were observed, in patients with *PALB2* and *ATM* mutations, respectively [60]. However, no difference in PFS was seen between patients with *ATM* variation as compared to those who were *ATM*-wildtype [60]. Further, retrospective studies suggest that pathogenic *ATM* mutations may be prognostic in pancreatic cancer, rather than predictive of therapeutic sensitivity [61]. Consistent with these findings, in patients with prostate and breast cancer, mutations in *ATM* and *CHEK2* are associated with minimal clinical activity of PARPi monotherapy [62,63,64].

The association between *STK11* mutations, of which germline variants drive Peutz-Jeghers syndrome, and PARPi sensitivity remains relatively unexplored [65]. *STK11* codes for LKB1, a serine-threonine kinase, which plays a role in DNA damage repair [66]. Further, 4–6% of pancreaticobiliary cancers show inactivation of *STK11* either by somatic mutation or LOH in the setting of a germline variant [67]. Cells deficient in *STK11* show impairment in HR and increased sensitivity to PARPi in vitro [66,68]. The PARPi, talazoparib, is being studied in combination with avelumab (anti-PD-1 monoclonal antibody) for the treatment of patients with *STK11* mutant non-small cell lung cancer (ClinicalTrials.gov Identifier: NCT04173507). Given the frequency with which *STK11* mutations are found in pancreatic cancer, and the potential sensitivity of these tumors to PARPi, inclusion of these patients in clinical trials assessing the use of platinum chemotherapy and PARPi is warranted.

Redundant and compensatory DNA repair protein functions represent a major challenge in the therapeutic targeting of non-*BRCA* HR mutations. Thus, next-generation treatment strategies are focused on combined targeting of multiple DNA repair proteins to overcome bypass mechanisms. For example, although pre-clinical studies suggest the sensitivity of *ATM* mutant pancreatic cancer to PARPi, as discussed above, this has not been seen clinically. One potential explanation for these observations is that compensatory ATR activity can mitigate any HRD phenotype in *ATM* mutant cancer [69]. However, dependency on ATR is a therapeutic vulnerability. To this end, co-targeting of PARP and ATR shows activity in vitro and in vivo in *ATM*-deficient pancreatic cancer models [70]. An ongoing trial assessing an oral ATR inhibitor in combination with olaparib for advanced solid tumors (including *ATM* mutant pancreatic cancer) will be informative (ClinicalTrials.gov Identifier: NCT03682289).

### 2.2. Additional Intrinsic Determinants of HRD Phenotype

The sensitivity of HRD tumor cells to platinum chemotherapy/PARPi is influenced by a number of cell intrinsic mechanisms beyond pathogenic HR gene mutations. For example, an in vitro CRISPR screen in *BRCA2* mutant cells demonstrated broad cellular processes to impact sensitivity to PARPi, including post-translational modification and drug transport [71]. Overall, important cell intrinsic mechanisms that modulate the HRD phenotype include (i) stabilization of replication forks, (ii) restoration/bypass of HR function and (iii) drug activity modification.

In addition to playing a central role in HR, BRCA1 and BRCA2 also function to stabilize stalled replication forks [72,73]. In support of this, in BRCA-deficient cells, stalled replication forks are acted upon by nucleases, such as MRE11, in an unrestricted manner, leading to excessive resection and ultimately fork collapse [74,75]. Further, molecular mechanisms that result in replication fork stabilization in *BRCA* mutant cells are associated with resistance to platinum chemotherapy and PARPi [76]. For instance, inhibiting the recruitment of MRE11 to stalled replication forks via genetic ablation of the MLL3/4 complex protein PTIP is associated with replication fork stabilization and resistance to DNA-damaging therapy [76]. Similarly, loss of the nucleosome remodeling factor CDH4 or the methyltransferase EZH2 eliminates excess DNA degradation via replication fork stabilization and induces resistance to platinum chemotherapy and PARPi, respectively [76,77,78]. Protein complexes involved in replication fork remodeling, such as SMARCAL1, also mediate MRE11 resection activity, and thus loss of SMARCAL1 is associated with platinum chemotherapy and PARPi resistance [79]. Taken together, these findings highlight replication fork stability as an emerging pathway of resistance to platinum chemotherapy and PARPi in *BRCA* mutant cancer.

Mechanisms of HR restoration/bypass are frequently unique to the underlying HR gene variant. Given the role of BRCA1 in end resection, *BRCA1* mutant cells are particularly susceptible to reconstitution of resection as a mechanism of HR restoration. In this regard, genetic deletion of 53BP1, a protein involved in NHEJ and DNA-end protection, reverses sensitivity of *BRCA1* mutant cells to platinum chemotherapy and PARPi [80,81,82]. Mechanistically, in *BRCA1* mutant cells, 53BP1 deletion is associated with a shift from NHEJ towards HR- and ATM-dependent processing of DSBs [81]. Several additional factors are downstream of 53BP1, including RIF1 and REV7, which enact critical resection inhibitory functions [83,84,85]. Further, the shielden complex, which contains REV7 and is a member of the 53BP1-RIF1-REV7-shielden pathway, similarly functions to restrain resection and is found to be decreased in PARPi-resistant PDX models [86,87]. Another protein of interest, DYNLL1, functions, at least in part, via direct binding to MRE11, limiting nuclease activity, and loss of DYNLL1 is associated with resistance to platinum chemotherapy/PARPi in BRCA1-deficient cells [88]. Distinct mechanisms of HR bypass are also seen in BRCA2-deficient cells. For example, loss of the histone acetyltransferase, KAT5, leads to increased NHEJ capacity via enhanced 53BP1 binding to DSBs and results in resistance to PARPi [71]. This highlights how differential modulation of the same pathway can have a distinct impact on sensitivity to platinum chemotherapy and PARPi depending on the specific HR gene variant. Another common mechanism which regulates HR proficiency, independent of a specific *BRCA* gene mutation, is loss of proteins that suppress RAD51 expression. Overexpression of RAD51 can reconstitute HR, bypassing the requirement for BRCA activity [89,90]. Regulators of RAD51 expression include HUWE1, a ubiquitin ligase, and E2F7, a transcription factor with a repressive function. Loss of either is associated with RAD51 overexpression and resistance to PARPi [71,91].

Several PARPi-specific resistance mechanisms exist, providing examples of drug-specific modulation of the HRD phenotype. To this end, overexpression of multidrug resistance protein 1 (MDR-1), a drug efflux pump with broad substrate specificity, is associated with resistance to PARPi [92]. MDR-1 is expressed in pancreatic cancer and is associated with poor outcomes when elevated [93,94]. Similarly, a number of transport proteins are required for efficient cellular uptake of platinum chemotherapy, and alterations in function can impact the efficacy of treatment [95]. Another interesting mediator of PARPi efficacy involves PARylation homeostasis. Poly(ADP-ribose) glycohydrolase (PARG) counteracts PARP by performing dePARylation, and loss of PARG is associated with resistance to PARPi in *BRCA2* mutant cells [96]. Finally, as occurs with most targeted cancer therapy, resistance to PARPi can develop via point mutations in the target protein. As such, *BRCA1* mutant cells expressing PARP1 mutants that lack DNA binding functionality are resistant to PARPi, likely due to the inability of PARPi to trigger PARP-DNA complex formation [97].

One potential approach to address the complexity and heterogeneity in tumor intrinsic control of DNA repair may be a personalized medicine approach to the treatment of HRD cancer. For example, in the future, comprehensive profiling of a panel of resistance pathways may guide patient-specific administration of rationally targeted combination therapy (e.g., PARPi plus ATM inhibition in 53BP1 mutant cancers). Additionally, as more patients with HRD pancreatic cancer are treated with platinum chemotherapy/PARPi, special attention will need to be paid to defining the clinical relevance of the alternative tumor intrinsic mechanisms of resistance we have described here.

### 2.3. Extrinsic Determinants of HRD

Non-malignant cells in the tumor microenvironment (TME) also play a role in shaping HRD. Pancreatic cancer is characterized by a hostile TME, consisting of dense fibrosis, immunoregulatory stromal cells, robust myeloid cell infiltration and a paucity of T cells [98]. Moreover, features of the TME play key roles in regulating sensitivity to chemotherapy, radiotherapy and immunotherapy [99,100]. The balance and geography of myeloid and lymphoid elements in the TME is a critical element which associates with clinical outcomes [101,102]. For example, a high ratio of CD8 T cells to CD68^+^ macrophages is associated with prolonged OS, while increased myeloid cell infiltration or close proximity of macrophages and tumor cells is associated with poor OS [101,102]. Consistent with the concept of immune infiltrates in the TME modulating sensitivity to platinum chemotherapy/PARPi, in unselected patients with breast cancer, robust pre-treatment T cell infiltration is associated with improved response to olaparib [103]. Tissue hypoxia also plays a role in regulating HR proficiency. In some instances, hypoxic tumor regions, which commonly occur in solid tumors, show decreased expression of HR proteins and increased sensitivity to chemotherapy [104,105]. However, the relationship between hypoxia and HR proficiency is dynamic, as severe hypoxia is associated with sensitivity to PARPi, while moderate hypoxia is associated with resistance [106].

#### 2.3.1. Interplay of the Tumor Immune Microenvironment and HRD

A bidirectional relationship between dysfunctional DNA damage repair (DDR) and the TME is increasingly recognized, where pathogenic mutations in HR genes drive distinct TME composition and TME members can modulate HRD. Cancer inflammation is central to this relationship. Cancer-associated inflammation involves recruitment of immunoregulatory myeloid cells to the TME, driving tumorigenesis and therapeutic resistance [107,108]. Further, initiation of cancer-associated inflammation often originates via tumor intrinsic mechanisms, such as through mutant *KRAS* programs [109]. In line with this, pathogenic *BRCA1, BRCA2* and *PALB2* mutations contribute to distinct tumor inflammatory architecture [110,111]. Elegant studies using human tissues and mouse models highlight the interplay between *BRCA1*, *BRCA2* and *PALB2* mutations and the TME. For example, *BRCA1* mutations in breast and pancreatic cancer are associated with increased density of tumor-associated macrophages (TAMs) and T cells, as compared to wildtype tumors and *BRCA2* mutant tumors [110,112]. Further, in mouse models of pancreatic cancer, *BRCA2* mutations are associated with an immune ‘desert’ phenotype, while *PALB2* mutant models show intermediate infiltration by immune cells [110]. Surprisingly, though, in a pan-cancer analysis, tumors harboring *BRCA2* mutations, as compared to those with *BRCA1* mutations, appear to be more sensitive to immunotherapy [111]. A detailed multi-omics assessment of *BRCA1* and *BRCA2* mutant mouse models of breast cancer revealed that this difference in sensitivity to immunotherapy is driven, at least in part, by the quality of the immune infiltrate. Immune infiltrates in *BRCA2* mutant tumors assume a potent anti-tumor phenotype, whereas myeloid cells infiltrating *BRCA1* mutant tumors show immunosuppressive capacity [111]. Further, TAMs, which are increased in *BRCA1* mutant tumors, are biologically active, inhibiting PARPi activity in *BRCA1* mutant breast cancer mouse models [112]. To this end, depleting TAMs, using colony-stimulating factor 1 receptor (CSF1R) blocking antibodies, enhances the activity of PARPi in *BRCA1* mutant mouse models of breast cancer [112]. TAMs are well-established to mediate resistance to chemotherapy in pancreatic cancer [113,114]. Taken together, HR gene mutations can influence the immune composition in the TME, and immunosuppressive cells in the TME can influence treatment sensitivity. Thus, detailed assessment of immune cells infiltrating HRD pancreatic cancer has the potential to reveal therapeutic strategies to enhance sensitivity to platinum chemotherapy/PARPi through targeting of the TME. Furthermore, targeting the TME to modulate HRD will be context-dependent, as distinct inflammatory responses are seen in *BRCA1*, *BRCA2* and *PALB2* mutant tumors.

#### 2.3.2. Interplay among PARP Inhibition and the Immune System

Growing evidence for a dynamic relationship between PARPi and inflammation in the TME further highlights the importance of tumor extrinsic factors in mediating the response to treatments [115]. A relatively small but persistent group of patients with *BRCA*-related pancreatic cancer demonstrate complete and durable responses with PARPi treatment [58,59]. This type of prolonged tumor control is reminiscent of durable responses to immunotherapy [116]. Further supporting this observation, mouse models of *BRCA1* mutant breast and ovarian cancer show that PARPi treatment, in addition to mediating cytotoxicity via synthetic lethality, triggers T cell-dependent anti-tumor immunity [117,118]. Underlying the immune-activating potential of PARPi is the cyclic GMP–AMP (cGAMP) synthase (cGAS)— stimulator of interferon genes (STING) pathway [117,118]. Treatment with PARPi triggers accumulation of excess cytosolic dsDNA, which is recognized by the DNA sensor cGAS [54,119]. cGAS initiates a signaling cascade, triggering STING activation and interferon production by tumor cells and dendritic cells, ultimately supporting anti-tumor T cell recruitment [54,117,118]. However, the immune-activating potential of PARPi may be context-dependent. For example, tumor cells that express the ectonucleotidase ENPP1 degrade extracellular cGAMP, whose breakdown products can be further metabolized to adenosine, an immunosuppressive metabolite, toggling the cGAS-STING pathway from immunostimulatory to immunosuppressive [120]. Furthermore, PARPi treatment is associated with upregulation of the checkpoint molecule programmed death ligand 1 (PD-L1) [121]. Given the immunomodulatory potential of PARPi, numerous clinical trials evaluating PARPi plus immune checkpoint blockade are ongoing (ClinicalTrials.gov Identifier: NCT03404960, NCT04666740, NCT04548752). Engaging productive anti-tumor immunity is especially challenging in pancreatic cancer, which is highly immunosuppressed. To this end, development of novel strategies which release and harness the immunostimulatory capacity of PARPi will be key to revealing the potential of PARPi treatment for pancreatic cancer.

## 3. Identifying HRD in Pancreatic Cancer

As detailed in this review, multiple tumor intrinsic and extrinsic factors influence whether a tumor will be sensitive to platinum chemotherapy/PARPi treatment. Furthermore, current methods of selecting patients for treatment, such as by germline *BRCA* mutation, are insufficient to fully capture the correct subpopulation(s) of patients who will derive clinical benefit from platinum chemotherapy/PARPi treatment [9]. Thus, ongoing efforts are focused on developing novel strategies to identify HRD.

### 3.1. Clinical Biomarkers

Although rudimentary, clinical characteristics can be used to identify patients with the potential to respond to platinum chemotherapy/PARPi. Germline mutations in *BRCA* genes are commonly associated with familial cancer syndromes characterized by increased risk of breast, ovarian and pancreatic cancer and other solid tumors [122]. Thus, a personal or family history of HRD-associated cancers has been explored as a surrogate for HRD. Additionally, in patients treated with platinum chemotherapy, evidence of a response is a clinical predictor of future treatment success with PARPi.

#### 3.1.1. Family History of Cancer

The biological impact of *BRCA1* and *BRCA2* mutations are histology-dependent, in that they are indispensable founding events in some tumors (e.g., breast, ovarian, prostate and pancreatic cancer) and inert in others [35]. Additionally, *BRCA* mutant breast, ovarian, prostate and pancreatic cancers, which are strongly associated with heritable germline *BRCA* mutations, are more likely to show sensitivity to PARPi as compared to *BRCA* mutant tumors of another histology [35]. Thus, family history, tumor histology and genotype intersect to modulate the clinical impact of HRD [33]. Patients with pancreatic cancer, and unknown HR gene status, who have three or more relatives with a history of breast, ovarian or pancreatic cancer, have prolonged survival with platinum-based treatment as compared to patients without a family history of *BRCA*-associated cancer [123]. However, it is not clear if selecting patients by family history of cancer identifies clinically relevant patients beyond those that would be identified by germline and somatic mutational analysis. For example, patients with pancreatic cancer without an HR gene variant, but with a strong personal or family history of *BRCA*-associated cancer, have no apparent clinical benefit from olaparib monotherapy, based on a recently published clinical trial [60]. Further, a phase II trial studying gemcitabine and oxaliplatin for the treatment of patients with metastatic pancreatic cancer and a family history (breast, ovarian, prostate or pancreatic) or a personal history (ovarian, breast or prostate) of cancer also showed little clinical efficacy, although HR mutational analysis was not reported, limiting conclusions [124]. Taken together, family history of malignancy is mostly a surrogate for established HR gene alterations, which are currently best identified by germline and somatic genetic analysis.

#### 3.1.2. Platinum Sensitivity

Given that platinum agents and PARPi induce HRD tumor cell cytotoxicity via shared mechanisms, platinum sensitivity can be used as a biomarker of response to future treatment with PARPi [125]. Ovarian cancer highlights this concept, where the clinical benefit of PARPi is highest in platinum-sensitive disease and lowest in platinum refractory disease [126,127]. Further, the association between platinum sensitivity and PARPi responsiveness is seen regardless of *BRCA* status in ovarian cancer [128,129]. As we highlighted in prior sections, patient selection using platinum-sensitivity plus *BRCA* or *PALB2* mutation status identifies a subset of patients with pancreatic cancer who benefit from PARPi maintenance [58,59]. Further, in patients with pancreatic cancer, regardless of HR gene mutation status, PFS to olaparib monotherapy is longer in platinum-sensitive disease as compared to platinum-resistant disease [60]. Together, these findings form the basis for two ongoing clinical trials evaluating PARPi plus checkpoint inhibition for the treatment of patients with platinum-sensitive pancreatic cancer with or without a *BRCA* mutation (ClinicalTrials.gov Identifier: NCT03404960, NCT04666740). Overall, platinum-sensitivity paired with HR gene profiling improves the identification of HRD in pancreatic cancer.

### 3.2. Laboratory Biomarkers

Despite the diversity of determinants modulating HR proficiency, all factors ultimately converge upon the same phenotype. Thus, current laboratory-based strategies are focused on identifying genomic or biochemical alterations that are proximal to any of the HRD determinants that we have discussed. Next, we highlight emerging laboratory-based testing strategies that have the potential to enter clinical use. We will focus on (i) testing for stereotyped genomic features and (ii) using functional assays to identify HRD (Table 2).

#### 3.2.1. Stereotyped Genomic Features

HRD results in genomic instability and the accumulation of distinct genomic alterations [125]. Specifically, (i) gross chromosomal abnormalities, or DNA ‘scars’, and (i) mutational signatures are characteristic of HRD. Thus, interrogation of genome integrity using DNA microarrays or next-generation sequencing (NGS) can be used to identify genomic patterns associated with HRD, potentially guiding patient-specific treatment decisions. Of note, the study of genomic scar and mutational patterns for use as predictive biomarkers has largely been established in patients with ovarian and breast cancer.

##### DNA Scars

Three major types of chromosomal aberrations are characteristic of HRD, including telomeric allelic imbalance (TAI), loss of heterozygosity (LOH) and large-scale transitions (LSTs) [130,131,132]. In patients with breast cancer, all three measures closely associate with the presence of *BRCA* mutations, and TAI is associated with improved response to platinum chemotherapy [133,134]. LSTs are increased in patients with HR gene mutant pancreatic cancer, but their presence does not necessarily associate with clinical outcomes [43].

Combining measurements of TAI, LOH and LSTs yields robust biomarkers for identifying clinically relevant HRD tumors. For instance, in patients with triple-negative breast cancer undergoing neoadjuvant platinum-based chemotherapy, a high ‘HRD score,’ generated by unweighted summing of TAI, LOH and LST, is associated with better pathologic response at resection, regardless of *BRCA* mutation status [134]. Additionally, this strategy forms the basis for an FDA-approved test called the myChoice HRD CDx assay (Myriad). This assay combines testing for pathogenic *BRCA* mutations with a Genomic Instability Score (GIS) based on TAI, LOH and LST, and is a companion diagnostic to determine eligibility of patients with ovarian cancer for treatment with niraparib or olaparib [135,136]. In patients with pancreatic cancer, GIS shows a sensitivity of 91% and specificity of 83% for identification of HRD, and higher GIS is associated with improved clinical outcomes with platinum chemotherapy treatment [9]. One benefit of GIS is that it can be performed on formalin-fixed paraffin-embedded (FFPE) tissues. However, as discussed next, alternative HR classifiers—ones that require whole genome sequencing from fresh frozen tissue—may have superior performance as compared to GIS. Another limitation of genomic scar-based techniques is that chromosomal alterations persist despite the development of resistance to platinum chemotherapy/PARPi and thus are not a dynamic marker of HRD [137].

##### Mutational Patterns

Reliance on low-fidelity DNA repair mechanisms to manage the inherent genomic instability of cancer, such as occurs in the absence of HR, facilitates the accumulation of distinct single-base substitutions (SBS), insertion deletions (indels) and rearrangements [138,139,140]. Further, detailed pan-cancer mutational analysis has revealed stereotyped mutational patterns that associate with cancer type and DDR deficiency state [139]. For example, an SBS pattern, termed signature 3, is associated with large deletions (up to 50 bp) with overlapping microhomology at rearrangement breakpoints [141]. Increased microhomology is seen in the setting of HRD due to the activity of alternative DNA repair pathways [142]. Further, *BRCA1*, *BRCA2* and *PALB2* mutant pancreatic cancer frequently shows signature 3 [42,143], and the presence of signature 3 is predictive of response to platinum chemotherapy in patients with advanced pancreatic cancer [9]. Additionally, in breast cancer, rearrangement signatures also associate with specific *BRCA* mutations. For instance, rearrangement signature 5 (RS5) is associated with *BRCA1* and *BRCA2* mutations, while rearrangement signature 3 (RS3) is associated with *BRCA1* mutations but not *BRCA2* mutations [144]. Similar associations between RS3/RS5 and *BRCA1/2* mutations are also seen in pancreatic cancer [9].

In addition to using individual signatures as clinical tools, investigators have leveraged the power of next-generation sequencing (NGS) to develop scores inputting multiple mutational patterns. The prototype of this approach is HRDetect, a weighted model of mutational signatures [145]. HRDetect accurately detects *BRCA* mutations and HRD in breast, ovarian and pancreatic cancer [145]. Further, application of HRDetect to a cohort of unselected patients with pancreatic cancer revealed several intriguing findings [9]. First, HRDetect outperformed GIS and signature 3 in its ability to identify HRD pancreatic cancer. Second, of patients with pancreatic cancer without a germline HR gene mutation, high HRDetect scores are present in up to 10% of patients. This subset of patients is frequently found to have somatic HR gene mutations, yet up to 30% of cases are unexplained by genomic analysis. Third, high HRDetect scores are predictive of improved survival in patients treated with platinum chemotherapy, regardless of HR gene mutation [9]. A challenge in implementing HRDetect clinically is the need for fresh frozen tissue, as FFPE-associated artifacts may prohibit the use of archived tissues [145]. This limits the current utility of HRDetect in clinical practice.

#### 3.2.2. Functional Assays

Given the dynamic nature of HR proficiency, static testing may be insufficient to fully clarify tumor HR status. One approach to deal with the conditionality of the HRD phenotype is the use of functional assays, which test the proficiency of HR ex vivo. Although functional assays are currently investigational, those testing the formation of RAD51 foci are intriguing. RAD51 forms a nucleoprotein filament at the site of DNA damage, promoting homologous DNA search and strand invasion, and is thus a critical downstream mediator of HR [22]. Moreover, absence of RAD51 foci formation has been shown to identify both *BRCA* mutant and wildtype HRD breast cancers and is predictive of response to platinum chemotherapy and PARPi treatment [146,147,148,149]. However, there remain clear challenges to the clinical application of RAD51-based assays. For instance, RAD51 foci are infrequently found in pre-treatment FFPE tissues [147]. Thus, assessment of post-treatment biopsies or fresh tissue stimulated ex vivo with DNA-damaging therapy (e.g., irradiation) is required based on the particular assay [147,150]. One strategy has been to co-stain FFPE tissues obtained 24 h post-chemotherapy, using immunofluorescent microscopy for RAD51 and geminin, a marker of S and G2 cell cycle phases. Co-staining with geminin allows for correction of RAD51 foci based on proliferation differences among tumors [146,147]. Several logistical issues are raised by this approach, including how to obtain multiple and on-treatment biopsies, how to institute standard tissue processing protocols when fresh tissue is required and how to develop reproducible pathology reading and reporting protocols. Finally, functional assays have not yet been validated in pancreatic cancer, further limiting the applicability. Even with these challenges, dynamic markers of HRD will be critical moving forward and further development of functional assays is needed.

### 3.3. Current Limitations and Next-Generation Strategies in Testing for HRD

The major limitations of current testing strategies for HRD include (i) lack of multi-feature assessments, (ii) non-standardized functional tests and (iii) insufficient dynamic testing. Overall, we suggest that the development of novel biomarkers is needed to improve identification of patients who will most benefit from platinum chemotherapy/PARPi treatment, with a focus on multi-feature assessment. For this to occur, advancements in technologies (e.g., functional testing, tissue acquisition, frozen tissue processing) will be necessary. Several emerging testing strategies that have been applied in other areas of cancer are a model for potential use in the identification of HRD. For example, the feasibility of an ex vivo tumor fragment platform to predict response to PD-1 blockade was recently established [151]. In this approach, tumor fragments are embedded in an artificial matrix and cryopreserved prior to characterization using multi-omics techniques, or are perturbed and analyzed to assess response to treatment [151]. Ex vivo tumor assessment for HRD could incorporate orthogonal testing, including functional assessment, interrogation of TME immune status and NGS. An alternative strategy for multi-feature assessment could include application of deep learning and artificial intelligence to histology specimens in order to identify tumors displaying HRD, as has been used to determine tissue of origin of cancers of unknown primary [152,153]. These techniques show promise for identifying germline *BRCA* mutant breast cancer based on histological features [154]. Another challenge in pancreatic cancer is difficulty obtaining sufficient tissue for testing, especially longitudinally. These cases would significantly benefit from further development of liquid biopsy approaches to identify HRD. To this end, the potential exists for using circulating tumor DNA or circulating tumor cell assays to assess mutational signatures or chromosomal instability in a non-invasive manner [155]. Taken together, technology advancements will support refined precision medicine approaches for patients with pancreatic cancer.

## 4. Inducing HRD

**Figure 2:**
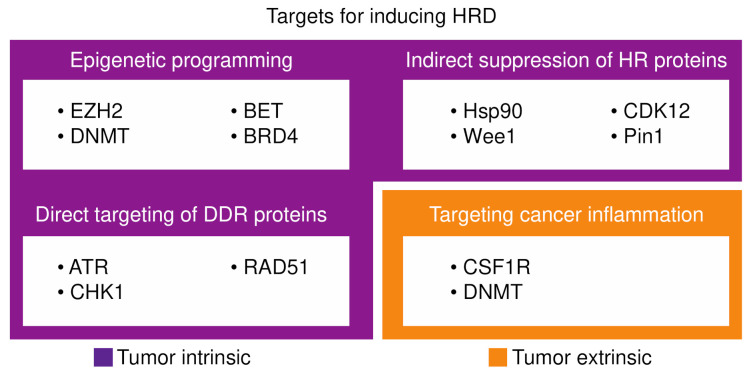
Targets for inducing homologous recombination deficiency (HRD).

Despite excitement around HRD as a target in cancer, the benefits of platinum chemotherapy/PARPi currently remain restricted to a minority of pancreatic cancer patients. Improved understanding of determinants of HRD will provide the opportunity for rational development of therapies aimed at inducing this phenotype. For example, as highlighted earlier, targeting the tumor immune microenvironment (e.g., depleting TAMs) may be a useful method to modulate sensitivity to platinum chemotherapy/PARPi. Several additional strategies are being investigated to extend the benefits of platinum chemotherapy/PARPi to a broader patient population. Approaches include: (i) epigenetic programming, (ii) targeting of DDR proteins and (iii) indirect suppression of HR machinery (Figure 2).

### 4.1. Epigenetic Programming of HRD

Targeting the epigenome is an attractive method for reprogramming cellular processes. In this regard, epigenetically targeted agents show significant potential for inducing HRD. For example, inhibition of EZH2, which plays a role in histone methylation, modulates cells to preferentially use NHEJ in the repair of DSBs and sensitizes HR-proficient ovarian cancer to PARPi in preclinical models [156]. Consistent with a role for targeting methylation, in vitro studies using non-small cell lung cancer (NSCLC) cells show that DNA methyltransferase inhibition (DNMTi) suppresses the expression of Fanconi anemia-related genes, leading to decreased RAD51 foci formation and heightened sensitivity to irradiation [157]. Intriguingly, DNMTi-induced HRD also intersects with the immune system. In fact, DNMTi, in part, drives HRD via innate immune activation and release of inflammatory cytokines, which suppress HR [158]. DNMTi also sensitizes breast and ovarian cancer cells to PARPi in vitro and in vivo [159]. A clinical trial studying the DNMTi decitabine in combination with the PARPi talazoparib for the treatment of acute myeloid leukemia is ongoing (ClinicalTrials.gov Identifier: NCT02878785).

Additional epigenetic regulators of interest are the bromodomain and extra-terminal domain (BET) family members (BRD2, BRD3, BRD4, BRDT) [160]. Studies in HR-proficient mouse models of cancer show that BET inhibition, specifically targeting of BRD2 and BRD4, sensitizes tumors to PARPi by suppressing RAD51 transcription [161,162]. Furthermore, inhibition of BRD4 is synthetically lethal with PARPi in multiple mouse models of cancer, including HR-proficient pancreatic cancer [163]. An ongoing clinical trial testing an oral BRD4 inhibitor as monotherapy and in combination with olaparib for the treatment of advanced treatment refractory solid tumors will provide insight into the potential of epigenetic targeting to induce HRD in patients (ClinicalTrials.gov Identifier: NCT03205176).

### 4.2. Targeting DNA Damage Repair Proteins

Attempts have been made to induce HRD by directly inhibiting DDR proteins. Earlier in the review, we discussed the concept of targeting ATR in *ATM* mutant cancers [70]. Moreover, ATR inhibition may also have a role in the treatment of *ATM* wildtype tumors. For example, in HR-proficient xenograft models of pancreatic cancer, the combination of ATR inhibition, PARPi and radiation results in synergistic anti-tumor activity [164]. Further, based on an early phase clinical trial, the combination of ATR inhibition and carboplatin shows activity in platinum/PARPi refractory ovarian cancer and *BRCA* wildtype solid cancers [165].

Another protein involved in HR initiation, CHK1, is a target of interest [166,167]. Based on pre-clinical work, inhibition of CHK1 sensitizes HR-proficient pancreatic cancer to chemotherapy and radiation [168,169]. The CHK1 inhibitor prexasertib combined with PARPi also shows activity in the treatment of patients with PARPi-resistant *BRCA* mutant ovarian cancer [170]. Although an early-phase trial failed to show the benefit of CHK1 inhibition in combination with gemcitabine for the treatment of patients with pancreatic cancer, in retrospect, the use of non-platinum chemotherapy limits conclusions about the effectiveness of CHK1 inhibition to induce HRD [171]. An ongoing challenge in pairing CHK1 inhibition with chemotherapy (e.g., FOLFIRINOX or gemcitabine cisplatin) or PARPi are the dose-limiting hematologic toxicities that emerge with combination therapy [170,172].

There is great interest in direct inhibition of RAD51, given its proximal role in HR. However, how to mitigate disruption of the physiologic role of RAD51 in normal tissues is a major limitation [173]. Intriguingly, a first-in-class RAD51 inhibitor shows promising pre-clinical results in combination with PARPi in vitro [174] and is actively being evaluated as a single agent for the treatment of B-cell malignancies and solid tumors (ClinicalTrials.gov Identifier: NCT03997968).

### 4.3. Indirect Suppression of Homologous Recombination Proteins

Modulating HR machinery by targeting proteins which intersect with HR has also been pursued as an avenue to induce HRD. A major target of this strategy, although one which has not yet generated robust clinical activity, is the chaperone protein Hsp90, which prevents proteasomal degradation of BRCA1 [175]. Hsp90 can be targeted using small-molecule inhibitors or can be suppressed using histone deacetylase inhibition, which leads to hyperacetylation of Hsp90, resulting in disruption of chaperone–client interactions [176,177]. Hsp90 inhibitors are generally well-tolerated as single agents [178]. However, early-phase trials do not suggest significant clinical benefit of monotherapy for the treatment of chemo-refractory pancreatic cancer [179] and Hsp90 inhibition does not appear to sensitize pancreatic cancer to gemcitabine [180]. Furthermore, in a phase III study, Hsp90 inhibition failed to augment the benefit of chemotherapy in patients with advanced NSCLC [181]. Hsp90 in combination with platinum chemotherapy produces profound hematologic toxicity, limiting the clinical utility of this combination [182]. Whether newer generation Hsp90 inhibitors, which target specific Hsp90 isoforms, will advance the field remains to be seen [183].

More promising is targeting of proteins involved in cell cycle regulation, which often also regulate DNA repair function. Wee1, a kinase which phosphorylates and represses Cdk1 activity, has dual roles in regulating entry into mitosis and maintaining HR proficiency [184]. In preclinical work, Wee1 inhibition sensitizes HR-proficient pancreatic cancer to chemotherapy and radiation [185,186,187]. Furthermore, Wee1 inhibition reduced Cdk1 phosphorylation in patients and shows significant potential as a radio-sensitizing strategy in combination with gemcitabine for the treatment of patients with locally advanced pancreatic cancer [188]. Wee1 inhibition in combination with PARPi shows potential activity in advanced solid tumors, yet is also associated with significant hematologic and GI toxicity [189].

Several additional investigational strategies for indirect targeting of HR have only yet been assessed pre-clinically. Within the cycle-dependent kinase class, Cdk12 and Cdk13 are known to regulate transcription of HR genes. Moreover, in pre-clinical work, inhibition of Cdk12 and Cdk13 represses HR proficiency and sensitizes tumors to platinum chemotherapy and PARPi treatment [190]. Additionally, inhibition of Prolyl Isomerase Pin1, which normally functions to limit degradation of BRCA1, sensitizes tumors to PARPi [191,192].

One common limitation to therapeutic strategies aimed at inducing HRD is high rates of hematologic and GI toxicity. Moving forward, the development of less toxic therapies with reduced off-target effects, coincident with improvement in treatment-specific supportive care, will ultimately enhance the potential of inducing HRD in clinical practice.

## 5. Conclusions and Future Perspectives

The identification of a subset of patients with HR gene mutations highlights the potential for personalized medicine in pancreatic cancer. In this regard, the FDA approval of maintenance olaparib represents a paradigm shift in the treatment of pancreatic cancer. However, only a minority of patients are found to harbor genetic alterations in HR genes, and even patients with HR-mutated disease almost inevitably experience cancer progression, despite current treatment strategies. HRD is defined by stereotyped mutations in HR genes and exquisite sensitivity to platinum chemotherapy/PARPi treatment. Importantly, HRD both overlaps with *BRCA* mutant tumors (although incompletely, as not all *BRCA* mutations are associated with sensitivity to platinum chemotherapy/PARPi) and extends beyond them. The HRD phenotype is dynamic, as it can be lost in the face of therapy or induced via experimental treatments in tumors without innate sensitivity to DNA-damaging therapy. Further, the HRD phenotype is driven by a confluence of genetic, tumor microenvironment and clinical factors. Given the complexity of HRD, integration of non-redundant testing strategies, such as combining evaluation of immune infiltrate composition, HR function (e.g., RAD51 foci formation) and gene signatures, may be needed to accurately identify patients. However, next-generation testing will need to be rapid and feasible in the clinical setting. Given exceedingly high response rates to platinum chemotherapy, early identification of HRD is critical for appropriate selection of both first-line treatment for advanced disease and neo-adjuvant treatment for early-stage pancreatic cancer. Overall, the development of novel strategies aimed at testing for and inducing HRD may ultimately allow for the expansion of a distinct and clinically relevant subset of patients with pancreatic cancer.

## Figures and Tables

**Table 1:** Determinants of clinically relevant homologous recombination deficiency.

Determinant	Mechanism	Platinum Sensitivity	PARPi Sensitivity	Notes
Primary determinants *				
HR gene mutations				
*BRCA1*	Abnormal end resection and invasion	↑	↑	
*BRCA2*	Abnormal strand invasion	↑	↑	Germline *BRCA2* mutations make up ~70% of *BRCA* mutations in pancreatic cancer
Non-*BRCA* HR genes	Variable loss of HR	↑ or ←→	↑ or ←→	*RAD51C/D* and *XRCC2* alterations may drive HRD in pancreatic cancer; Germline *ATM* and *CHEK2* are not associated with signatures of HRD in pancreatic cancer
Secondary reversion mutations	Frame-shift mutation allows translation of active truncated protein	↓	↓	
Secondary determinants^#^				
Replication fork stability				
CDH4	Loss leads to replication fork stabilization via reduced resection	↓	↓	Clinical relevance in pancreatic cancer remains to be determined
EZH2				
PTIP				
SMARCAL1				
HR reconstitution/bypass				
53BP1-RIF1-REV7-shielden	Loss leads to reduced NHEJ and release of end resection antagonism	↓	↓	Specific to *BRCA1* mutant cancers
DYNLL1	Loss drives reduced inhibition of MRE11-mediated end resection			
RAD51	Overexpression increases HR proficiency			
Drug specific alterations				
Drug transport	Excess drug efflux or inefficient drug uptake	↓	↓	P-glycoprotein/MDR1 is frequently overexpressed in pancreatic cancer
PARP1 point-mutations	Reduced DNA binding capacity prevents PARP trapping			
dePARylation	Loss of PARG reconstitutes PARylation activity			
Tumor microenvironment				
Tumor infiltrating T cells	PARPi drives cGAS-STING activation and T cell anti-tumor immunity	-	↑	Pre-treatment T cell infiltration associated with improved response to PARPi in breast cancer
Tumor associated macrophages	-	-	↓	Anti-CSF1R enhances PARPi activity in *BRCA1* mutant breast cancer models
Tissue hypoxia	Suppression of RAD51 and BRCA2 translation	↑↓	↑↓	Severe hypoxia is associated with supression of HR, while moderate hypoxia promotes resistance to PARPi

* Primary determinants are required; #Secondary determinants modulate clinical phenotype.

**Table 2:** Testing for homologous recombination deficiency.

Target	Definition	Diagnostic techniques	Clinical tests	Benefits	Limitations	Notes
Genomic scars						
Telomeric allelic imbalance (TAI)	Subchromosomal regions of allelic imbalance extending to the telomere	SNP array; Array-based comparative genomic hybridization; Next-generation sequencing	Genomic instability score (GIS) an unweighted sum of TAI, LOH and LSTs	Can be performed on FFPE tissues	GIS has reduced sensitivity and specificity for identifying HRD as compared to mutational signatures; Scars persist regardless of change in HR proficiency	LST are increased in HRD pancreatic cancer, but may not be associated with response to platinum chemotherapy
Loss of heterozygosity (LOH)	Regions of LOH of moderate size (greater than 15 Mb but less than the entire chromosome)					
Large scale transitions (LST)	Chromosomal break between two regions of at least 10 Mb					
Mutational signatures						
Signature 3	Large deletions (up to 50 bp)	Whole genome sequencing	HRDetect a weighted model incorporating mutational signatures	Signature 3 and HRDetect have high sensitivity and specificity for detection of HRD in pancreatic cancer	Requires fresh tissue due to FFPE associated artifacts; Signatures persist regardless of change in HR proficiency	RS3 is associated with *BRCA1/2* mutations and RS5 is associated with *BRCA1* mutations
Rearrangement signatures (RS) 3 and 5	Tandem duplications <10kb (RS3) or deletions <100kb (RS5)					
HR function						
RAD51 foci	RAD51 foci arise during nucleoprotein filament formation	Immunofluorescence (IF) microscopy	Various, including assessment of *ex vivo* irradiatiated or post-chemotherapy biopsy specimen using IF microscopy	Dynamic marker of HR proficiency	Some assays require on-treatment biopsy due to limited RAD51 staining in pre-treatment FFPE specimens; IF microscopy is challenging to scale for clinical application	Established in breast cancer; Validation in pancreatic cancer needed

## Data Availability

Data sharing is not applicable to this article.
